# Monkeypox 2022 outbreak in non-endemic countries: Open questions relevant for public health, nonpharmacological intervention and literature review

**DOI:** 10.3389/fcimb.2022.1005955

**Published:** 2022-09-20

**Authors:** Maria Rosaria Capobianchi, Antonino Di Caro, Chiara Piubelli, Antonio Mori, Zeno Bisoffi, Concetta Castilletti

**Affiliations:** ^1^ Department of Infectious Tropical Diseases and Microbiology, Istituto di Ricovero e Cura a Carattere Scientifico (IRCCS) Sacro Cuore-Don Calabria Hospital, Verona, Italy; ^2^ Saint Camillus International Medical University, Rome, Italy

**Keywords:** monkeypox, prevention measures, seroepidemiology, subclinical infection, animal reservoir, spillover, zoonosis, orthopoxvirus

## Abstract

Starting from mid-May 2022, cases of human monkeypox started to rise in several non-endemic countries. By mid-July, more than 17000 confirmed/suspect cases have been reported by at least 82 countries worldwide, with a regular incremental trend. In order to contain the disease diffusion, risk evaluation is crucial to undertake informed decisions and effective communication campaigns. However, since orthopoxvirus infections so far have attracted low attention, due to the eradication of smallpox 40 years ago, and to the confinement of human monkeypox almost exclusively to endemic areas, several unresolved issues concerning natural history, ecology and pathogenesis remain. To this respect, we identified some open questions and reviewed the relevant literature on monkeypoxvirus and/or related orthopoxviruses. The results will be discussed in the perspective of their relevance to public health decisions, particularly those related to non-pharmacological interventions.

## Introduction

Since May 7, 2022, countries across Europe, the Americas, and Australia began to report monkeypox (MPX) disease cases in individuals that had no travel links to endemic areas in Africa. With little epidemiological information available, researchers began to question the reason for the rapid rise in cases.

**Figure 1:**
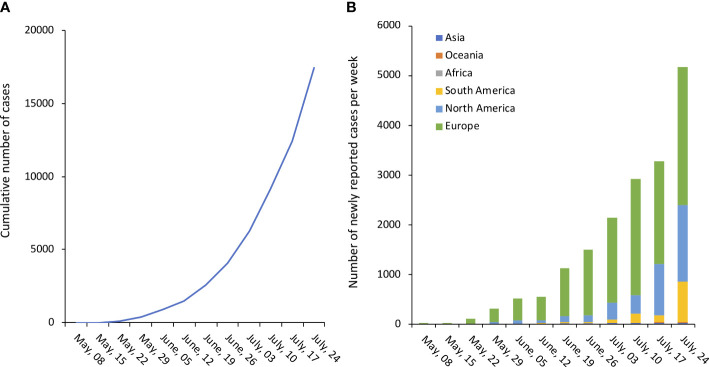
Number of confirmed monkeypox cases. **(A)**. Cumulative number of confirmed cases worldwide. **(B)**. Number of newly reported confirmed cases per week, by continent/subcontinent. Source of original data https://www.monkeypox.global.health/, accessed on July 25, 2022.

By mid-July, more than 17000 confirmed/suspect cases have been reported to World Health Organization (WHO) from at least 82 countries, with a steady incremental trend as showed in **Figure 1** (Global.Health). On July 23^rd^ 2022, WHO Director-General Tedros A. Ghebreyesus declared the escalating global monkeypox outbreak a Public Health Emergency of International Concern (WHO).

MPX is endemic in over 11 countries of Equatorial Africa (WHO) where hundreds of cases of human MPX cases are detected annually. In these countries the monkeypoxvirus (MPXV) is transmitted to humans from either wild animals or, more rarely, from infected humans. More in details, in about 70% of cases of human MPX the virus was contracted from animals serving as natural virus carriers (mostly rodents, but monkeys are also involved); in about one-third of such cases it was acquired from infected humans (CDC, 2003).

Close interpersonal contact is likely the means of human-to-human transmission. The reported infectivity of humans with MPX for close contacts so far has been somewhat lower (12.3%) than in smallpox, for which it varies from 37% to 88% (Marennikova, 1989).

In the present outbreak, widespread human-to-human transmission is involved, and cases in Europe and North America have occurred mostly in men who have sex with men (MSM) who attended recent mass gathering events, aged 20–50 year, thus mostly not vaccinated against smallpox. Whether MPXV is sexually transmitted requires further careful study. As a matter of fact, clusters of viral infections can occur in any group of persons in close contact, and promiscuous behavior during mass gathering events may cause increased risk of exposure to affected body areas.

To date, all publicly available MPXV genomes from the 2022 outbreak belong to the West African lineage, which may cause less severe disease and has a lower case-fatality rate than the Congo Basin clade. The genomes from the 2022 MPX outbreak show low genetic distance (0.4-1.5 nucleotide changes/genome), typical of strains belonging to the same transmission chain, and share a common ancestor with MPXV from Nigeria 7/28/22 4:47:00 PM.

The 2022 MPXV branch diverges from the related 2018-2019 viruses by a mean of more than 50 SNPs, which is far more (roughly 6-12 fold more) than one would expect considering previous estimates of the substitution rate for orthopoxviruses (OPXVs) (1-2 substitutions per site per year) (Firth et al., 2010). Such a divergent branch might represent accelerated evolution, and the accumulated mutations are consistent with the action of host APOBEC3, so that this feature can be considered a sign of potential MPXV human adaptation in ongoing microevolution. (Isidro et al., 2022).

The origin of the ongoing global MPXV outbreak is still unknown; it is likely that the initial infection (case 0) has been imported from an endemic country according to the usual pattern and the virus started to circulated among close (eventually sexual) contacts until the number of cases and the unusual body location of lesions claimed attention on the unexpected findings. Indeed, there is growing evidence that cross-continent, cryptic human transmission has been ongoing for longer than previously thought (Virological.org; Gigante et al., 2022).

**Figure 2:**
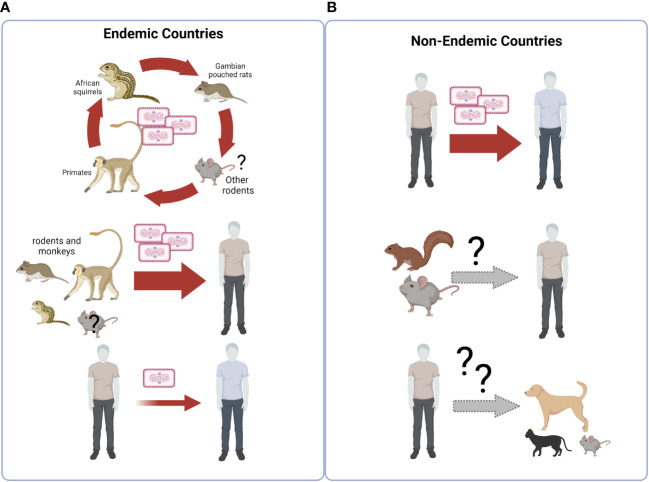
Ecology of Monkeypoxvirus. **(A)** The virus is endemic in over 11 countries of Equatorial Africa, where its circulation is primarily maintained through spread among sylvatic animals, including primarily rodents such as rope squirrel, tree squirrel, Gambian pouched rat and dormice. Monkeys can also be infected. Occasional transmission to humans occurs from animals encountered during hunting, preparation and consumption of bush meat, forest exploration and agricultural activities. Human-to-human transmission is less frequent. **(B)** In non endemic countries, an outbreak of human MPX cases is currently going on, characterized by sustained human-to-human transmission, occurring through intimate contact with body (including genital) secretions, cutaneous lesions, respiratory droplets and contaminated fomites. Transmission from humans to sylvatic/peri-domestic rodents, or to domestic pets, has not been documented so far, but could represent a potential risk for MPXV endemization in the fauna of new countries outside of Africa. Created with BioRender.com.

The biological, environmental, behavioral, and social reasons for this unexpected increase are unexplained so far, and require urgent definition through a unifying, One Health approach. To this respect, it is important to consider that bidirectional flow of MPXV transmission is possible at the human-animal interface, and MPXV transmission from humans to susceptible animals may trigger animal transmission chains that can get out of control and establish endemicity in wild animals, see **Figure 2**.

Overall, understanding the dynamics of viral transmission in the context of continuously evolving epidemiological pattern is indispensable for preventing further spread of MPXV, and, ultimately, for a coordinated approach to epidemic preparedness and response.

In fact, although third generation vaccine and anti-OPXV drug are now available, the stockpiling for such medical countermeasures is limited. Hence, non-medical interventions are crucial to intercept the infection spread along the pathway of transmission and amplification in humans and at the human-animal interface, to prevent the establishment of endemicity in new territories and, possibly, in wild animals (Zumla et al., 2022).

In this paper, we identified some open questions, and reviewed the relevant literature on MPXV and/or on related OPXVs to address these questions. The results have been discussed in the perspective of their relevance to public health decisions, including those related to non-pharmacological intervention.

The questions are the following.

1. Is MPXV virus transmissible from affected humans to animals, i.e. pets, and how likely is this event? Which animals are likely to be susceptible? Could this event ignite transmission cycles and promote virus endemization among sylvatic animals in western countries?
2. Does the asymptomatic carrier state for MPXV occur? Does infectious virus shedding from different anatomical sites occur from infected persons and how long does it last after healing?
3. Is the actual diffusion of MPXV in the human populations from endemic areas known? Are seroprevalence studies feasible? Which laboratory methods are available and affordable?

## Methods

To examine peer reviewed scientific articles relevant for the formulated questions, Internet search engines (Google, Google Scholar, PubMed, Medline, and Ovid) were queried with appropriate keywords as search terms, consistent with each single question. The study period was 1970 to 2022. The articles reviewed included disease surveillance studies, seroprevalence studies, review articles, case reports, systematic reviews, series and disease outbreak investigations. Recent relevant articles concerning the current MPX outbreak, not peer reviewed, were included for an updated information. Non–English-language articles were excluded from review. The selected methodology met all recommended criteria for narrative reviews, including several keywords, use of 2 or more Internet search engines, a defined study period, and article inclusion and exclusion.

## Results

### Question 1

Is MXPV transmissible from affected humans to animals, i.e. pets, and how likely is this event? Which animals are likely to be susceptible? Could this event ignite transmission cycles and promote virus endemization among sylvatic animals in western countries?

### Data from the literature

There are numerous examples of new pathogens introduced by intentionally or accidentally imported animal species and became established in native animal population (e.g., Yersinia pestis introduced by rats to the western United States) (Azad, 2004). Although the transmission from animals to humans of several OPXVs (Carletti et al., 2009; Cardeti et al., 2011; Puro et al., 2018; Lapa et al., 2019) and of MPXV in particular (Arita and Henderson, 1976; Breman et al., 1980; Arita et al., 1985) has been well documented, only few reports indicate that transmission of OPXVs from humans to animals can naturally occur. Most of these reports date back to the time of the smallpox elimination campaign, when the possibility that the virus could be transmitted to monkeys (nonhuman primates, NHP) was the subject of intense investigation. Already in 1841 Anderson described a vesicular exanthem in monkeys in Panama and, later (1922), Bleyer reported a similar outbreak in *Cebus* monkeys in Brazil (cited in Noble and Rich, 1969). Both epizootics caused widespread death in the monkey population and preceded, or were coincident with, smallpox epidemics in humans from nearby villages. However, the aetiology of these epizootics could not be confirmed by laboratory testing, unavailable at the time, and the signs and symptoms described seem quite different from those observed in experimental infection of monkeys (Noble and Rich, 1969). Potential transmission of smallpox to monkeys by fomites has been indicated in 1874 by Zuelzer, who reported that smallpox-infected materials contained in a wire basket and placed with healthy monkeys transmitted the infection to these animals (cited in Noble and Rich, 1969).

Experimental infection in NHP models has been described, in particular Noble and Rich demonstrated both the airborne transmission and the possibility of multiple transmission cycles (up to six) in *Macaca irus* (Noble and Rich, 1969). Despite its susceptibility to variola and its ability to transmit the infection, no naturally occurring cases of smallpox have ever been reported in this species, nor in related species such as *Macaca mulatta*, a species that occurs throughout the whole Asia, suggesting that even if accidental spill over from human to animal really occurred, it did not trigger the endemization of smallpox in monkeys. Now the relevant question is, “*are we sure that this will also happen in the event of transmission of MPXV to animals outside of Africa*?”

The animal to animal transmission of MPXV occurs systematically (Reynolds et al., 2010; Orba et al., 2015) and signs of increased circulation in a wild chimpanzee sentinel system have been recently reported (Patrono et al., 2020), contradicting the initial observations that highlighted the extreme rarity of the infection, at least among NHP (Arita et al., 1972). A substantial circulation of OPXV among wild African NHP has been detected also by serological investigations (Breman et al., 1977); as a matter of fact, animal-to-animal transmission is “still” the main route for maintaining virus circulation in endemic countries (Patrono et al., 2020).

Some insight in the animal-human virus transmission flow can be inferred from the outbreak of human MPXV cases occurred in US in 2003 (Reed et al., 2004). Animal-to-animal transmission (from giant Gambian rat imported from Africa to local pets) was indeed responsible for the initial events triggering the outbreak that involved more than 40 peoples in several US locations. Fortunately, in that case the MPXV transmission to the North American native animal species, i.e. the black tailed prairie dogs (*Cynomys ludovicianus*), did not initiate the virus spread among the wild fauna, so that local endemization did not follow that episode, or at least there is no evidence of it. This notwithstanding, more than 300 exposed animals at risk to be infected escaped the surveillance system established at that time (Bernard and Anderson, 2006). Subsequent investigation, however, highlighted the susceptibility of cotton rat (a rodent common in Mexico) and prairie dogs to this infection (Knight, 2003). Prairie dogs were also adopted as animal model for testing vaccines and therapies (Xiao et al., 2005; Hutson et al., 2011). Although other animal species were found to be infected, the human cases were epidemiologically linked only to contact with prairie dogs (Bernard e Anderson 2006), substantiating the suspected role of this local rodent as amplifying host, that could have played a crucial role for the development of the epidemic (Reynolds et al., 2010a).

On the basis of genetic evolution and number of genes dedicated to host species adaptation included in their genomes, OPXVs have been proposed to fall into one of three host-utilization categories: i. highly specialized (single-host); ii. broad host range; iii. ‘cryptic’; including those OPXV species whose host is very poorly characterized (Reynolds et al., 2018). For the number of host genes in its genome and its biological properties, MPXV is included, like cowpox (CPXV) and vaccinia viruses (VACV), in the “broad host range” category (Reynolds et al., 2018).

As for the animal host spectrum, the potential natural sources of MPXV outside of Africa remain unknown (Haddad, 2022). A recent review of animal species susceptible to MPXV infection can be found in Silva et al., 2020. Numerous ecologic and serological investigations have suggested that not only NHP (also of non-African origin), but numerous other animal species are susceptible to the infection, in particular rodents such as rope squirrel, tree squirrel, Gambian pouched rat and dormice (Marennikova et al., 1972; Marennikova & Seluhina, 1976; Hutson et al., 2015). However, the virus has been so far isolated only twice from wild animals: once from the rope squirrel (*Funisciurus anerythrus*), Zaire, in 1985 (Khodakevich et al., 1987), and once from the sooty mangabey (*Cercocebus atys*), Côte d’Ivoire, in 2012 (Radonić et al., 2014).

We did not find any documented evidence of livestock being infected with MPXV. Regarding transmissibility to domestic animals or pets such as dogs and cats, although these are susceptible to infection with other OPXV and can play a relevant role in human infection (Carletti et al., 2009; von Bomhard et al., 2011; Lapa et al., 2019), we have not found any description of natural or experimental infection with MPXV in these animals. In particular cats, especially those only partially domestic, if susceptible and potentially infectious, might be able to spread the infection to wildlife, i.e. to rodents that escaped their ambushes. To this respect, conflicting data are reported on the susceptibility of peridomestic rodents (*Mus musculus* and *Rattus rattus)* to MPXV, but probably the difference is due to different development status of the studied animals i.e. new-born vs adult (Marennikova & Seluhina, 1976; Reynolds et al., 2018). As a matter of fact, anti-OPXV antibodies compatible with previous MPXV infection have been detected in the peri-domestic roof rats (*Rattus rattus)* in Uganda (Salzer et al., 2013), a MPXV free country, suggesting a potential niche very close to human habitats of MPXV or a similar OPXV.

### Take home message

The broad spectrum of infections demonstrated by OPXVs similar to MPXV (e.g. CPXV and VACV) (Guagliardo et al., 2020; Silva et al., 2020) can reasonably make us to predict that, as a result of the spread in the wildlife, MPXV would eventually find susceptible hosts and a suitable niche for its spread even outside of Africa, unless local circulation of other OPXV(s) provides an unfavourable background due to cross-protective immunity.

The recent discovery of a new OPXV in Europe (Gruber et al., 2018) indicates our insufficient knowledge about the biodiversity of existing viruses of this genus (Babkin et al., 2022). Investigation aiming to improve our knowledge of their spread in wildlife, particularly in wild rodents (Kinnunen et al., 2011; Fischer et al., 2020), could help to assess the risk of MPXV endemization.

Invasion of new niches of animal wildlife by MPXV would represent the starting point of a very challenging situation, enhanced by the fact that transmission may be silent because infected animals usually do not show the same visible symptoms as humans (Quarleri et al., 2022), hindering the adoption of adequate preventive measures.

Therefore, the hypothetical risk of human-to-animal transmission cannot be overlooked, and appropriate measures, such as physical distancing from animals (including domestic pets) and proper waste management should be put in place to prevent the virus from being transmitted from infected humans to susceptible animals (including pets) at home, in zoos and wildlife reserves, and to peri-domestic animals, especially rodents (WHO).

### Question 2

Does the asymptomatic carrier state for MPX occur? Is atypical clinical presentation common? Does infectious virus shedding from different anatomical sites occur from infected persons and how long does it last after healing?

### Data from the literature

Transmission of OPXV from infected hosts (either human or animal) that show little or no or even atypical symptoms has been recognized as an important issue yet >30 years ago, since the lack of symptoms precludes the adoption of precautions to avoid contact with infectious sources. (S. Marennikova et al., 1985).

Literature data cover infections from MPXV and other OPXVs in humans as well as in animals.

Evidence of subclinical infection mostly derives mainly from retrospective serological investigation, but virus detection methods have also been applied.

In humans, the first relevant data date back to the seventies, when the most relevant OPXV was smallpox, before its eradication. A study based on a low sensitivity method for virus detection, i.e. virus isolation, showed that about 9% of family contacts of smallpox cases shed infectious variola virus in oral secretions, mainly from unvaccinated persons (Sarkar et al., 1974). Consistent results were obtained by retrospective serological investigation conducted in an African village where a recent smallpox outbreak occurred. In this study, the frequency of serologically positive persons was about twice that of overtly diseased persons (i.e. 27.3% vs 14%), suggesting substantial frequency of inapparent infections (Heiner et al., 1971).

However, the transmission of infection from a mild variola form (variola sine eruptione) has not been documented (cited in Breman & Henderson, 2002).

Inapparent forms of human MPX have been recognized as early as in 1983, concerning outbreaks from former Zaire in the immediate post-smallpox eradication period (Jezek et al., 1983). In fact, antibody testing, covering the period 1980-1984 in the former Zaire, indicated 0.6% of contacts who had no evidence of clinical disease with positive serological results, consistent with past infection (Jezek et al., 1986). Subclinical infection with MPXV, involving mainly young persons, was also inferred from another serological investigation in the same area (Jezek et al., 1987).

More recent data came from studies conducted since the cluster of about 40 human cases of MPX observed in the US in 2003, ignited by exposure to infected prairie dogs that, in turn, became infected after contact with a giant Gambian rat imported from Africa. Serological signs of recent infection (IgM) were detected in an asymptomatic health care worker who assisted MPXV-infected patients (Fleischauer et al., 2005). Retrospective serological investigation identified several additional asymptomatic infections, mostly found in persons vaccinated against smallpox (Hammarlund et al., 2005; Karem et al., 2007).

In the ongoing worldwide MPX outbreak, three asymptomatic infections have been detected by retrospective screening of anorectal and oropharyngeal swabs collected in May 2022 from 224 individuals undergoing screening for sexually transmitted infection (STI) in a Belgian clinic; in all cases the infection was acquired from likely asymptomatic individuals, supporting the hypothesis of sustained unrecognized MPXV circulation in the target population (De Baetselier et al., 2022).

In Cameroon the serological status among the staff at a primate sanctuary where a MPX outbreak occurred in captive chimpanzees in 2016 and among residents from nearby villages was evaluated, showing a prevalence of anti-OPXV antibodies of 34.4%. Interestingly, the prevalence of IgG and IgM among asymptomatic persons too young to have received smallpox vaccination was 6.3% and 1.6%, respectively (Doshi et al., 2019).

Recent serologic evidence from Ghana showed OPXV infection to be frequent in rodents and humans with rodent exposure despite the absence of any reported human disease in the area (Reynolds et al., 2010a).

A human asymptomatic infection with a novel, ectromelia-related OPXV, occurring in monkeys from a nature reserve in Italy, has been described in an animal caregiver working into the reserve (Puro et al., 2018).

Concerning the occurrence and duration of viral shedding from different body sites, especially in the absence of symptoms, few data are available from past studies. Infectious virus has been cultured from the oral mucosa of a smallpox patient before the appearance of lesions (cited in Breman & Henderson, 2002), and, in fact, transmission of smallpox from patients during the incubation phase has been reported (Wehrle et al., 1970).

In recipients of VACV-based smallpox vaccine ACAM2000, virus shedding from the vaccination site after the fall of the crust was observed in up to 23% of vaccinated persons, and in 3.4% the virus shedding persisted for at least 6 weeks (Pittman et al., 2015).

Prolonged detection of viral DNA in blood and in the and upper respiratory tract has been observed in MPX cases occurred in UK between 2018 and 2021 (Adler et al., 2022).

In the present MPXV outbreak, prolonged shedding of viral DNA from multiple body sites, including the genital secretions, eventually accompanied by the presence of viral infectivity, has been reported (Antinori et al., 2022).

In animals, old studies performed with natural as well as non-lethal experimental infections show that asymptomatic virus carrier state may occur with MPXV as well as with other OPXV such as ectromelia cowpox and ratpox viruses (Shelukhina et al., 1979).

However, most recent information about kinetics of OPXV replication and shedding in animal models derives from lethal challenges (MPXV in non human primate models, and rabbitpox in rabbits), to evaluate the effect of potential antivirals. Hence all these infections are accompanied by severe clinical signs and do not address the issue of asymptomatic virus shedding (Hutson et al., 2021).

Few recent data, derived from farms with confirmed outbreaks caused by VACV, show that viral DNA and viral infectivity is present in milk, feces and blood not only from dairy cows with clinical signs, but also from asymptomatic animals (Rehfeld et al., 2017).

### Take home message

Inapparent shedding of infectious virus may represent a source of MPXV infection, especially mediated through respiratory and oral/genital secretion carrying the infectious virus.

Despite a snatchy body of evidence, all available studies, from either animal or human infections with either MPXV or other related viruses, indicate that inapparent virus shedding may occur in the following situations:

1. Asymptomatic or paucisymptomatic MPXV carriers, who are expected not to be rare;
2. Patients who are in the pre-symptomatic stage, where virus shedding from oral (and possibly other) mucosal sites in apparently healthy persons may be frequent;
3. Patients who get over the symptomatic stage, where prolonged virus shedding frequently occurs from healed skin lesions and can be found in oral (and possibly other) secretions.

For these reasons, it is crucial to establish appropriate information policy, addressing those settings that are considered most at risk of catching MPXV, such as mass gathering events or locations where intense and highly promiscuous sexual activity is expected to occur, in order to avoid at risk behaviours and to promote timely consultation of medical advice in case of signs of infection.

### Question 3

Is the actual diffusion of monkeypox in the human populations from endemic areas known? Are seroprevalence studies feasible? Which serological methods are available and affordable?

The establishment of the real extent of the MPXV diffusion is complicated by several issues.

The first is that the clinical appearance, in particular the bullous rash, of MPX is similar to that of chickenpox. As a matter of fact, it is not a surprise that several suspected MPX cases occurred during outbreaks in 2007 in MPXV endemic regions were, actually, caused by infection with varicella-zoster virus (MacNeil et al., 2009), challenging the effectiveness of rash illness surveillance.

Evidence of exposure to MPXV can be inferred by the frequency of antibodies against OPXV. There are no commercially available tests for OPXV, due to the low interest in commercial exploitation of such products, so far. Hence serological evaluation is almost exclusively based on in house methods, that have been developed and are available in a very limited number of laboratories. Most of estimates from Africa have been obtained from assays performed at a poxvirus laboratory at USA CDC in Atlanta, where blood samples have been shipped in a dry form at room temperature, after collection on filter strips (Nobuto blood filter strips, NBFS). Established methods include enzyme linked assays (ELISA), hemagglutination inhibition (HAI), radioimmunoassay adsorption (RIA), indirect immunofluorescence (IFA) and neutralization-based assays (Karem et al., 2005; Leendertz et al., 2017).

Generally, these methods do not allow to discriminate between different OPXV species within this genus, that show substantial cross-reactivity. More sophisticated assay (based on western blot) possess a little increased discriminatory power for antibodies against different OPXV species (Salzer et al., 2013) but are cumbersome and could not be applied for large scale field investigation. However, it is difficult to differentiate current infection from past exposure, on the basis of serologic results, particularly in the absence of detailed clinical and epidemiologic data. To this respect, IgM detection may be of support. As an example, in an epidemiologic and ecologic investigation performed in 2017 in a rural region of the Republic of the Congo, to assess the extent and the possible source of an MPX outbreak among the persons retrospectively classified as suspect cases, 100% turned out to be IgG-positive, and 88.9% were IgM-positive (Doshi et al., 2019).

Overall, the lack of standardized methods to measure anti OPXV antibodies represents a potent obstacle to the generalization of results that may hamper comparison from different studies.

The estimate of MPXV circulation within humans is hampered also by the fact that a variable proportion of the human population >50 years old had received the VACV-based vaccine before the universal smallpox vaccination was halted. Antibodies from vaccination recognize a small number of proteins shared with pathogenic virus strains, while recovery from infection also involves humoral responses to unique antigens present in the proteome of the infecting OPXV; nevertheless, the available assays do not allow to discriminate between antibodies elicited by vaccination and those induced by natural infection (Keasey et al., 2010).

Moreover, the estimates of anti OPXV antibody duration show consistent variation according to the laboratory method and the epidemiological setting that has been investigated. In several studies the vaccinated persons maintained a long-lasting post vaccination immune response (Taub et al., 2008; Leendertz et al., 2017). For instance, a study from Australia suggested that antibody responses can persist for decades even in the absence of natural boosting (Costantino et al., 2020), and a >20 years duration was consistently seen among 16 retrospective cross-sectional studies (Kunasekaran et al., 2019). In a study from Japan (Hatakeyama et al., 2005) very long duration of both binding and neutralizing anti-VACV antibodies in vaccinated people was observed. In fact, in this study the frequency of anti-VACV IgG measured by ELISA among subjects who were born before 1962, between 1962 and 1968, and between 1969 and 1975, was 91.0, 90.3, and 58.2%, respectively, showing substantial overlap with the presence of neutralizing antibodies. On the contrary, the U.S. Center for Disease Control suggests that anti OPXV immunity completely wanes after 5–10 years from vaccination (Hammarlund et al., 2003).

Another potential bias is represented by the fact that antibody prevalence might vary even between relatively close geographical areas (Reynolds et al., 2010b), and often samples are collected from spotted areas, not representing the whole territory.

With these bias in the background, the available serological data from African countries endemic for MPX will be reviewed, together with those from Brazil, characterized by intense OPXV circulation in humans as well as in domestic and sylvatic animals.

### Africa

In older studies, the overall seroprevalence against OPXV, estimated with RIA or HAI, was 16% in the Republic of Congo (Lederman et al., 2007) and 19% in Democratic Republic of Congo (Jezek et al., 1987).

Based on ELISA results, a serosurvey of antibodies to OPXV conducted among residents of Likouala region, Republic of Congo, where a MPX outbreak associated with nosocomial spread occurred in 2003, showed an overall IgG prevalence of 56.9%, with higher risk observed in persons living in forest galleries than in those in savannah, and among those living in areas where human MPX cases had occurred in the past compared with those living in other localities. IgM presence, indicative of recent exposure to OPXV, presumably MPXV, was observed in 1.7% of study participants. and was associated with older age, that in turn, could be linked to activities more likely to be performed by adults, i.e. hunting and managing animal carcasses. (Lederman et al., 2007).

In the same region, during an outbreak in 2017 on the basis of serological results several independent inter-human chains of transmission were identified, and *Cricetomys* giant pouched rats showed presence of OPXV antibodies, adding evidence to this species’ involvement in the transmission and maintenance of MPXV in nature (Doshi et al., 2019).

Data from seroprevalence studies conducted in 2010 in eastern Sierra Leone, where, by the time of assessment, no cases of human OPXV infections had been reported since 1986, suggest that, although likely infrequently, human exposure to OPXVs continues to occur in that region after the eradication of smallpox and in the absence of reported human infections in previous decades (Macneil et al., 2011).

High anti-OPXV seroprevalences (19 to 26%) have been observed in Central and Western Africa in previously smallpox-unvaccinated people, indicating regular contact with OPXVs, even in the absence of recognized outbreaks (Leendertz et al., 2017).

In Camerun the serological status was evaluated among staff at a primate sanctuary where a MPX outbreak occurred in captive chimpanzees in 2016 and among residents from nearby villages. The proportion of individuals positive to anti-OPXV antibodies was 34.4%. Evidence of OPXV exposure (IgG positive, 6.3%; IgM positive, 1.6%) was reported among some of those too young to have received smallpox vaccination (i.e. born after 1980), but these infections were asymptomatic (Guagliardo et al., 2020).

### Brazil

Evidence of OPXV circulation in Brazilian Amazon region, when no OPXV outbreaks were yet been reported (around 2010), was provided from seroprevalence studies, that indicate 27.89% in the overall population, and 23.38% in the non-vaccinated population, suggesting high exposure to OPXV in that region (Mota et al., 2010). VACV, which once circulated widely in Brazil, or unrecognized diffusion of a novel OPXV in this region were considered possible responsible for the high prevalence of anti-OPXV antibodies in humans, that was paralleled by similar frequency in animals like cattle, monkeys and rodents.

As a matter of fact, the evidence of intense animal circulation of OPXVs in rural areas of Brazil in the absence of recognized outbreaks was corroborated by subsequent serological studies in humans, dairy cattle, horses, and wild animals (Costa et al., 2016; Borges et al., 2017; Miranda et al., 2017; Rehfeld et al., 2017; Borges et al., 2018), and by outbreak reports in domestic buffalo calves from various Brazilian areas, caused mainly by the brazilian VACV strain, named Pernambuco (VACV-PE) (Franco-Luiz et al., 2016; Costa et al., 2016; Lima et al., 2018).

### Take home message

Monitoring human exposure to MPXV and other OPXVs is crucial to understand better the ecology of these viruses and their animal hosts and the dynamics of a possible re-emergence, and perhaps to predict future spill-over events.

Serological studies have been performed in the past and are currently performed in order to establish/confirm the circulation of OPXVs in a given population.

The available methods are non commercial, relatively difficult to perform, and are hampered by lack of standardization. Hence, comparison of data from different studies is difficult. In addition, available methods do not allow to identify with sufficient discriminatory power the virus species against which the antibodies are directed, so that the viral species involved in the various settings can only be indirectly inferred by concomitant epidemiological and direct virological evidence, that often are lacking.

Despite these challenges, the serological and related epidemiological data so far collected indicate that, in endemic countries, the diffusion of human OPXV infections reflects their frequency in domestic and peridomestic animals, or in animals that humans encounter in activities connected with hunting, preparation, and consumption of bush meat, as well as forest exploration and agricultural activities. On the whole, in endemic countries, humans do seem accidental hosts of MPXV, not relevant for their maintenance in the natural circulation.

Generally, the frequency of seropositivity in humans, as well as in animals, is higher than that expected on the basis of symptomatic manifestations, indicating large proportion of subclinical infections.

Overall, user friendly, standardized assays are required to solve this challenging issue, and laboratory capacity building is necessary in the endemic regions, in order to precisely map the circulation and host range of OPXV species in the present time.

## Discussion of results and relevance to public health decisions, including those related to non-pharmacological intervention

After about fifty years from the defeat of smallpox, as the immunity elicited by vaccination vanishes, another OPXV, the MPXV seems to have acquired the ability to be effectively transmitted from human to human and could occupy the ecological niche left free by the disappearance of smallpox, causing a considerably less severe disease. Whether this will happen will also depend on our ability to react, in particular it will depend on the countermeasures, medical and non-medical, that we will be able to put in place in an effort that can only be coordinated worldwide. Of course, some of the factors that contributed to the “defeat” of smallpox are now different for MPXV. Among them the absence of animal reservoirs for smallpox. The fact that MPXV is present in African wild fauna and the possibility that a reverse spill-over from humans to animals may occur in other continents certainly represents a major challenge. Hence the need for a global “One health” approach with increased animal surveillance, both to improve knowledge on animal reservoirs in Africa, and to quickly detect and try to contain any reverse spill over in animals from other continents. The invasion of new niches of animal wildlife in new continents represents a consistent risk of a widening the flow of zoonotic MPXV transmission, possibly enhanced by the fact that infection signs in animals may go unrecognized, preventing the implementation of prevention measures. Therefore, appropriate measures, such as physical distancing from animals (including domestic pets) and proper waste management, should be put in place to prevent human-to-animal virus transmission, in every setting where potentially susceptible animals (especially rodents) live (at home, in zoos and wildlife reserves).

Another relevant aspect is the need to promptly detect patients capable of transmitting the infection, so that containment and infection control procedures can be put in place. Although the diagnosis of OPXV infections has been improved compared to the 70s, thanks to the development of molecular tests, yet not widely commercially available, much remains to be done for antibody investigation, that remains crucial to establish the extent of diffusion, taking in mind the possibility of asymptomatic or atypical clinical presentations.

In particular, the available tests, mostly not commercially distributed, do not effectively distinguish, neither in humans nor in animals, the specific antibodies against MPXV vs those to other OPXVs and those elicited by currently available vaccines vs natural infection. Therefore, in order to carry out appropriate serosurveillance investigations, serological diagnosis must be improved, with particular emphasis on assay standardization, and appropriate funding to support research in this area must be allocated.

Concerning the identification of patients capable of transmitting the infection, the problem remains for those who can do it during prodromes, or during infections with subclinical or atypical clinical presentation; in addition, the possibility of prolonged release over time after healing should be taken in duly account. Also in this case, research efforts will be necessary, in order to improve our knowledge about the natural history of the infection, so that, based on improved understanding, we may establish timely and adequate countermeasures. The challenge is arduous, but, at least for the present time, the infection due to the MPXV strain involved in the current worldwide outbreak does not seem to be particularly severe. In fact, until now, the affected persons belong to a generally healthy segment of human population, but, if not halted, the risk is that other segments of the human population, more fragile, may be reached by the virus, with more severe consequences at individual level, and increased burden for the health systems of the affected countries.

## Author contributions

MC and AC: Conception of the study; formulation of the study aims and methodology. CC: study coordination, review of the literature and manuscript drafting. CP and AM: collection of literature data and manuscript drafting. ZB: overview of the study aims and methodology. All authors contributed to the article and approved the submitted version.

## Funding

This study has been partially funded by Italian Ministry of Health Fondi di Ricerca Corrente-L3P1 to IRCCS Sacro Cuore-Don Calabria Hospital

## Acknowledgments

We warmly thank Dr. Andrea Fittipaldo (IRCCS Sacro Cuore Don Calabria Hospital) for her contribution.

## Conflict of interest

The authors declare that the research was conducted in the absence of any commercial or financial relationships that could be construed as a potential conflict of interest.

## Publisher’s note

All claims expressed in this article are solely those of the authors and do not necessarily represent those of their affiliated organizations, or those of the publisher, the editors and the reviewers. Any product that may be evaluated in this article, or claim that may be made by its manufacturer, is not guaranteed or endorsed by the publisher.

## Abbreviations

OPXV, orthopoxvirus; MSM, men who have sex with men; MPXV, monkeypoxvirus; NHP, non human primate; STI, sexually transmitted infection; VACV, Vaccinia virus.
